# Mothers’ views on the school-starting process for deaf children

**DOI:** 10.3389/fpsyg.2025.1694936

**Published:** 2026-01-08

**Authors:** Necla Işıkdoğan Uğurlu, Nilay Kayhan, Sakine Hakkoymaz

**Affiliations:** 1 Department of Special Education, Faculty of Education, Zonguldak Bulent Ecevit University, Zonguldak, Türkiye; 2 Department of Speech and Language Therapy, Faculty of Health Sciences, Ankara Yıldırım Beyazıt University, Ankara, Türkiye; 3 Department of Elementary Education, Faculty of Education, Hasan Kalyoncu University, Gaziantep, Türkiye

**Keywords:** deaf children, early childhood, mother, inclusiveness, right to education

## Abstract

Early diagnosis, hearing aid implementation, education, and active family participation play an important role in hearing loss. Cochlear implantation, hearing support technologies, and auditory rehabilitation are essential components of early intervention. Difficulties experienced by families of children with hearing loss from birth may negatively affect the children’s development. This study aims to examine the early childhood school-preparation journeys of children with hearing loss through their mothers’ voices. Using a qualitative research method, this study was conducted with 20 mothers of children with hearing loss. Demographic information forms and interview questionnaires were used as data collection tools. Semi-structured face-to-face interviews were conducted with the mothers. The data were analyzed using content analysis. Four main themes emerged: “realization and guidance, family life changing with diagnosis and search for empowerment, holistic support for children with HL, and sustainable and accessible support mechanisms.” The mothers emphasized that newborn hearing screening plays an important role in early diagnosis and intervention and that they require informational, social, and financial support following their child’s hearing loss diagnosis. They reported that they receive most of their support from family members and from other families with children who have hearing loss, while also expressing a lack of broader social support. One of the most striking findings is that families need information and support not only on the implementation of hearing aids for children diagnosed early but also on the communication method to be used.

## Introduction

Hearing loss needs to be diagnosed early, and intervention should begin promptly (CDC, 2024). More than 5% of the world’s population, equivalent to 430 million people, requires rehabilitation to address hearing loss (including 34 million children). It is estimated that by 2050, more than 700 million people (or 1 in 10) will have hearing loss (World Health Organization, 2025). When necessary precautions are not taken, hearing loss can lead to more serious problems. If left untreatened, it affects many areas of life at the individual level. For example, difficulties in communication and speech, as well as cognitive skills, negatively affect children’s academic and social development, especially with respect to communication, language, and speech (World Health Organization, 2025). Therefore, a comprehensive intervention should be planned for deaf children from an early age; hearing assistive devices such as hearing aids and cochlear implants should be provided. The level of hearing loss should be taken into account in early intervention services (Dazert et al., 2020). Early diagnosis and implementation of devices enable positive development in children. It is well established that children who undergo early screening and cochlear implantation demonstrate better receptive and expressive language skills (Boons et al., 2013a). For deaf individuals, early diagnosis, the use of hearing aids, education, and family involvement play an important role (Tüfekçioğlu, 2003) The World Health Organization recommends investigating the causes of the increase in hearing loss and draws attention to the need for accessible hearing care in all regions of the World (World Health Organization, 2018).

Accordingly, early diagnosis, appropriate interventions, and inclusive practices are important. Utilizing educational services at the earliest possible time and including families in these services are critical for preventing potential difficulties (Alduhaim et al., 2020; Girgin and Kemaloğlu, 2017). Family-centered early intervention services are important because they empower families and facilitate collaboration with professionals (Moeller et al., 2013). Many studies on health and education services in developing countries are conducted to prevent/treat deafness, and these studies include families as well as children. Including families in the services and plans provided to their children is a critical dimension for minimizing the impact of the loss. Hearing loss needs to be monitored from the prenatal and postnatal periods to old age. Approximately 60% of hearing loss in children is preventable through public health measures. Early diagnosis and comprehensive interventions in hearing loss are important for both children and adults. Because hearing loss affects the families as well as the child, family participation in the auditory rehabilitation process is essential (World Health Organization, 2025). Education for deaf children has always been a matter of debate, and the regulations governing it are tied to countries’ education policies. Inclusive practices in health and education for deaf individuals offer them a qualified social life perspective (European Union of the Deaf, 2022).

Early education services include support services such as education, health, and care for children aged 0–6/8 who are at risk or have special needs (Bowe, 2008; Keilty, 2010). The main purpose of support services in early education is to improve children’s and families’ quality of life by minimizing the difficulties they experience and to prepare them for later life. Thus, early education services are significant for deaf children, as for all children. Today, studies on the quality and necessity of the education services provided to these children continue to increase (Howard et al., 2011). In Türkiye, as in the rest of the world, the type and level of infants’ hearing loss are determined, and they are directed to the National Newborn Hearing Screening Program (NNHSP) to benefit from health opportunities (Kemaloğlu, 2015). However, in Türkiye, as in many developing countries worldwide, families’ need for information in the fields of education and health arises with the introduction of newborn screening. Early diagnosis and intervention are critical for both the family and the child. However, difficulties in the planning and follow-up processes of intervention services have been reported (Campbell-Wilson, 2012). This situation is especially pronounced in families with low incomes and living in rural areas. Meeting families’ needs regarding their children in the evaluation, diagnosis, and treatment processes, and ensuring their cooperation with experts, are critical for their children’s rehabilitation. Cooperation with families and meeting their educational needs also facilitates children’s early use of hearing aids and their benefit from early education services. Due to the cooperation between families and experts who play a key role in supporting deaf children, the quality of home-based services has been improving. Thus, families spend more quality time with their children and support their development (Boons et al., 2013b). Children’s interactions with their families are particularly important, especially for language, communication, and social skills. Hence, holistic support mechanisms are needed in the early period both for children and families (Bronfenbrenner and Evans, 2000).

In the literature, it is reported that approximately 90%–95% of deaf children have no family history of deafness. Thus, families are concerned about how to interact with their children regarding language, communication, and social skills (Dirks et al., 2019). It is inevitable for families who have no knowledge or experience about their deaf children to experience feelings such as sadness, anxiety, and stress due to the difficulties they experience. Families experience difficulties in many areas, such as acquiring knowledge, sharing their experiences, and meeting the communicative purposes of their children, which support language and communication skills that play an important role in their children’s development (Kurtzer-White and Luterman, 2003). To overcome these difficulties, families must start to benefit from early education services when their deaf children are diagnosed early. The literature states that when families are informed and included in planning appropriate intervention services for deaf children, deaf children show development similar to that of their peers in language, communication, and social skills (Nittrouer and Burton, 2001). Thus, planning that involves the family and child together is particularly important in early childhood special education services.

Educational practices for deaf children must be planned without discrimination. They must be accessible and inclusive, based on equal opportunities (Levesque and Duncan, 2024). Special education services in early childhood are addressed in two stages: early special education (0–36 months) and preschool special education services (36–72 months) (Gül and Diken, 2009). Preschool education includes educational processes that support children’s physical, cognitive, language, communication, and social development and are appropriately tailored to children’s individual characteristics and needs in a planned and inclusive way (Cook et al., 2008). It is evident that educational services for children with special needs are supported, and there are legal regulations in this regard worldwide (Heward, 2013). The Individuals with Disabilities Education Improvement Act [IDEA], one of these legal regulations, legally states that children with special needs must benefit from special education services in the preschool period (Hardman et al., 2006). This regulation guarantees the education of children with special needs in the preschool period. When examining the legal regulations regarding children with special needs in Türkiye, preschool education has been adopted as a legal principle. Active participation of children and families in all stages of special education is emphasized (Milli Eğitim Bakanlığı/Ministry of National Education, MEB, 2018) The preschool education program in Türkiye supports children’s development from an early age, taking into account their developmental characteristics and individual learning needs. The program, prepared with an inclusive approach, is based on fundamental principles and is implemented through skill-based programs (Milli Eğitim Bakanlığı/Ministry of National Education, MEB, 2024).

Families are one of the main actors in these services. Family education should include not only the early childhood period but also various stages, including support services, preschool, institution changes, and transitions. Parents should be involved in supporting their children’s social and academic skills at home and in school, from the diagnosis stage through school transitions, follow-up, and the use of hearing aids, using technology effectively, and choosing the most appropriate communication methods (Coppens et al., 2012). Studies in the literature on deaf children in the preschool period are generally focused on the realization and diagnosis processes (Most and Zaidman-Zait, 2001). It is also evident that studies to determine families’ and children’s needs in the early childhood period, or those that holistically address children’s developmental levels, are limited (Fitzpatrick et al., 2008). The United Nations Convention on the Rights of Persons with Disabilities emphasizes an accessible and inclusive life. However, when appropriate arrangements are not made early for children with hearing loss, they are left behind from their earliest years (Deafness Resource Center, 2023; United Nations, 2006). In this study, we adopted an approach grounded in a comprehensive understanding and universal legal regulations. As stated in the United Nations resolutions, the Universal Declaration of Human Rights, and the International Covenants on Human Rights, human rights are inherent and inalienable rights. The foundation of this study is the philosophy of inclusivity and equal access to education as a social model. We focused on what deaf students and their mothers experience in a life of silence. We explored mothers’ narratives of their struggles to access education for their deaf children in early childhood. We examined the experiences of mothers of deaf children. The study covers the experiences of hearing-impaired students in Türkiye, including how they access early childhood special education and support services and the efforts of their families in this process. The problem of this study is that deaf children, who lack access to quality and comprehensive healthcare and education in early childhood, face more challenging conditions in the years to come for themselves and their families.

The research also focuses on support services and family experiences in early childhood special education of deaf students in Türkiye. Given this limitation in the literature, our main aim in this study is to examine mothers’ awareness of their children’s deafness, diagnosis, hearing aid implementation, and cochlear implantation, and their experiences with their children’s preparation for preschool education. Thus, this study sought to examine families’ experiences with the diagnosis, evaluation, and treatment of deafness in their children from birth. Moreover, the social and medical support units families applied to, and their experiences during the transition from home to institution-school, were examined. In this respect, the study focused on families’ experiences with the transition from home to a preschool education institution (the school where they receive formal education services with their peers) and on the processes of deaf children.

In the study, answers to the questions below have been sought:

(1) What are the life experiences of mothers of deaf children in the preschool education period regarding deafness from diagnosis to intervention?
(2) According to mothers of deaf children in the preschool education period, what are the needs of deaf children and their families?
(3) According to mothers of deaf children during the preschool period, what are the main sources of support for deaf children and their families?

## Materials and methods

The study aims to reveal the experiences of mothers of deaf children regarding the transition to preschool services, from the time of diagnosis through the parents’ perspectives. We developed the interview guide based on a literature review, the opinions of academic experts who support families’ rights advocacy for deaf children, and those of teachers with experience working with deaf children. When preparing the interview guide, we adopted a model that reflects mother–child interaction. The starting point of this research was the bioecological model, which examines children’s interactions with the people around them. The bioecological model is based on and developed from Bronfenbrenner’s ecological systems theory. According to this theory, children undergo biological, social–emotional, and cognitive development as they interact in different environmental contexts throughout their development (Bronfenbrenner and Morris, 2006; University of Washington, 2024). Based on this model, which seeks to understand children’s relationships and how they interact with their environment, we developed a protocol to examine the experiences and opinions of mothers with hearing-impaired children in the preschool period. This protocol aims to understand the processes of diagnosis, assessment, rehabilitation, education, and health care during the early childhood and newborn periods. Since these are experiences, we adopted a qualitative research design.

We designed the interviews as part of a qualitative research study, grounded in real-life experiences and reflecting personal opinions. When contacting institutions, we sought the opinions of teachers who graduated from higher education institutions and currently work with deaf children at the educational institution. To communicate with institution administrators, we leveraged the role of academic-scientific partnerships with the local government, municipality, and directorate of national education in the province where the third researcher works. The third researcher collaborates with local administrators on educational and social-cultural activities in the province where she works, organizing social-cultural training for families and children. She made appointments and met with the administrators of these two institutions. After receiving positive responses, she informed the families through the administrators of these institutions and the special education teachers who teach the classes.

As this study, conducted using a qualitative research method (Bogdan and Biklen, 2007), was grounded in parents’ experiences, a phenomenological design was employed (Moustakas, 1994; Yıldırım and Şimşek, 2013). While determining the participants, it was taken into account who would be included in the study and which age and diagnostic group the parents’ children would be in; accordingly, participants were selected using the purposive sampling technique (Yıldırım and Şimşek, 2013). In the study, the researchers asked parents whether they would participate voluntarily; only mothers agreed, so they were included as the only parent group. The criteria for participation were being in the preschool age range, having a clinical diagnosis of deafness, and accepting voluntary participation.

### Participants

In total, 20 typically hearing mothers of deaf children participated in this study. Mothers live in a provincial center in southeastern Turkey. All mothers reside in the same province. Mothers’ life experiences from diagnosis to intervention regarding their children served as the basis. For this purpose, the deaf children’s hearing history and families’ experiences regarding the realization, diagnosis, support services, and preparation for the preschool period were examined. The criteria for “clinical deafness diagnosis” were independent of features such as unilateral or bilateral hearing loss, degree, type, and age of onset (congenital or acquired). Mothers answered these questions during the interviews. Participating mothers signed the informed consent form.

### Data collection

The researchers developed the study plan, prepared the interview forms, and submitted ethics committee applications in August 2024. Ethics committee permission was obtained from the institution where the third researcher works; the data were collected through face-to-face interviews with the mothers at the children’s educational institutions between October and November 2024. The study was planned by three researchers. The first two researchers hold doctoral degrees in special education. They support the language, speech, and communication skills of children with special needs, including deaf children, and provide effective, evidence-based interventions in literacy and family education. The third researcher is a faculty member in classroom education. The third researcher conducts studies on teaching literacy to children during the preschool and primary school periods, on narration, and on supporting language and communication skills. After receiving ethics committee approval, the researchers approached officials at the preschools attended by the children. Two institutions providing support education services for deaf children in the Southeastern Anatolia Region of the country agreed to cooperate. First, the third researcher met with the institution managers, then explained the purpose and content of the study to the families. The family meeting attended by the parents was planned by the third researcher in the institutions where we conducted the first interviews. After the family meeting attended by the parents, interviews were scheduled with 20 mothers who responded positively to the information and consent forms.

After informing the parents in the first stage, 20 mothers who agreed to participate and would comply with the interview plan were selected for the study. We first contacted the administrators and authorized managers of the institutions to reach the mothers and fathers of all children attending them. The institutions’ authorities informed the children’s parents about the study and that we needed voluntary participants. Then, the researchers conducted family interviews using their written documents to explain the study’s purpose and the process to inform parents; not all families who received the information agreed to participate. We found that we could not reach more participants because mothers were unable to set aside time when they came to the home or institution. They had the burden of schooling, nutrition, and the care of their children with typical development. Moreover, some mothers did not want to meet even though they had time. We excluded them from the study in line with the principle of volunteer participation. The study included only mothers of children with hearing loss who voluntarily agreed to participate. Our goal was to reach more than 20 parents; however, after clarifying the work schedule, only 20 mothers had time to participate. We believe that mothers answered all questions sincerely during the interviews and that the themes were determined based on data saturation. The mothers participating in this study were interviewed, the purpose of the study was explained, and the form was introduced. The mothers responded to the demographic information and questions in writing. The interview was not recorded on audio or video for later transcription. The third researcher transcribed it simultaneously. The written data obtained from 20 mothers were made into a total whole.

### Data collection tools

The data collection tool is a semi-structured interview form. The data collection tools are provided in Annex-1-2-3. Four forms were used for data collection. These are the Information Form for Deaf Child, Information Form for Participant Mother, and Semi-structured Interview Form. The mothers were asked 13 questions about the child, 5 about the parents, 18 demographic questions, and 6 open-ended questions. The researchers triangulated the data. The third researcher visited the institution and conducted interviews with the administrator and special education teachers at the institution where the mothers’ children received supportive education services. She took interview notes on the diagnosis of their children, the start of education, the families’ expectations, and their reported participation in the education. She also kept observation notes about the environment. In this study, multiple data triangulations were conducted between the mothers’ responses and the other data mentioned above. In this study, data were collected only in accordance with the study purpose and ethical permissions. This was only analyzed in this study and constituted the findings. According to this declaration, all data are original research data. To protect the confidentiality of study participants, identifying information is not explicitly shared. The authors did not provide the supporting data. The results and analysis in the article are original.

### Data analysis

The content analysis method was used to analyze the data (Morgan, 1993). The mothers participating in this study were interviewed, and their responses were transcribed. Written data were coded, and themes were identified. The dataset consisted of 33 pages: 18 pages of demographic information and 15 pages of responses to open-ended questions. The first part of the data consisted of 1,342 paragraphs, 2315 lines, and 3,766 words. The researchers turned these data into tables (please see Tables 1–3). The tables included demographic information about the deaf children and their families. The second part of the data consisted of 15 pages, 742 lines, 2,285 words, and 397 paragraphs. These data are based on mothers’ experiences of the transition to preschool education for deaf children from diagnosis to intervention. A content analysis approach was adopted in the study (Creswell and Creswell, 2018). The data were coded by the first two researchers using the independent coding method, and categories were determined. After this step, differences and similarities among mothers’ answers were taken into account, and the dataset was organized into sub-themes and main themes. In the intercoder agreement percentage calculation for the first and second researchers, Mıles and Huberman's (1994) Reliability = [Agreement/(Agreement + Disagreement)] × 100 formula was used as the basis; the result was 93%.

**Table 1:** Demographic information regarding the families.

No	Mother’s education	Father’s education	Mother’s age	Father’s age	Father’s profession	Family’s source of support	Source regarding D	Perceived SEL
M1	Secondary School	High School	26	30	Employee	Husband and family members	Toy	Average
M2	High School	High School	31	35	Employee	No support	Education institution	Average
M3	Secondary School	Secondary School	26	32	Employee	No support	Audiologist, hearing aid company	Average
M4	Illiterate	Primary School	35	39	Employee	Education institution	Education institution	Low
M5	Primary School	Primary School	26	35	Technician	Family, educational institution	Educational institution, toy	Low
M6	Illiterate	Primary School	35	42	Employee	Family	Internet, hospital	Low
M7	Primary School	Secondary School	29	39	Employee	Husband	Hospitals, educational institutions	Low
M8	High School	High School	43	58	Retired	Special education teacher	Education institution	Low
M9	University	University	35	38	Doctor	Grandmother	Toy, book	Average
M10	Secondary School	Secondary School	22	27	Employee	Family, hospital staff	Educational institution, toy	Good
M11	Primary School	Secondary School	48	51	Employee	No	Teacher, book	Low
M12	Primary School	Secondary School	30	32	Farming	Family, educational institution	Teacher, expert	Good
M13	Primary School	Secondary School	40	45	Driver	No support	Internet, books	Good
M14	High School	High School	38	38	Employee	Education institution	Toy, books	Average
M15	Primary School	High School	32	35	Employee	Education institution	Educational material	Average
M16	High School	High School	27	34	Employee	Husband, mother	Teacher, expert	Good
M17	Secondary School	Secondary School	28	32	Employee	Education institution	Story, cartoon	Average
M18	Secondary School	Secondary School	24	32	Furnisher	Grandmother, grandfather	Internet, experts	Average
M19	Secondary School	Secondary School	28	32	Employee	Husband, educational institution	Storybooks, cartoon	Average
M20	Primary School	High School	43	40	Driver	Education institution	Stories, toys	Average

Education Institution: Special Education and Rehabilitation Center M: Mother SEL: Socioeconomic Level.

**Table 2:** Demographic information regarding deaf children (information was obtained through interviews with mothers).

Code	Child gender/age		Mother’s source of support	Family’s number of children (except D)	Support needs of deaf child
M1	Female	4-year-old	Husband	2	LSC
M2	Male	4-year-old	No Source of Support	1	LSC
M3	Male	4-year-old	No Source of Support	2	LSC, preparation for school
M4	Male	4 years 6 months	SEI	4	LSC
M5	Male	5-year-old	SEI	2	LSC, peer interaction
M6	Male	4 years 4 months	No Source of Support	1	LSC, game skills
M7	Male	4 years 11 months	Hospital, Special Edu. Tchr.	1	LSC
M8	Female	5-year-old	Special Edu. Tchr.	1	LSC
M9	Female	5-year-old	SEI	1	LSC
M10	Male	4 years 11 months	Own family members	0	LSC
M11	Male	5-year-old	No	4	LSC
M12	Male	4 years 11 months	SEI	2	LSC
M13	Female	4 years 2 months	SEI	6	LSC
M14	Male	5-year-old	SEI	2	LSC
M15	Female	5 years 4 months	SEI	1	LSC
M16	Male	5 years 3 months	SEI	1	LSC
M17	Male	5 years 1 month	SEI	1	LSC
M18	Male	5 years 6 months	Husband, family members	1	LSC
M19	Male	5-year-old	Teacher	1	LSC
M20	Female	5 years 5 months	Doctor, SEI	3	Academic skills, LSC

Special Education Institution: an education institution responsible for special education support services under the name of the Special Education and Rehabilitation Center; Language, Speech, and Communication Skill: LSC. When examining the support resources of mothers whose children are mostly between the ages of 4 and 5, it is noteworthy that they receive support from special education and rehabilitation support centers. The support needed is focused on their children’s language skills.

**Table 3:** Children’s use of hearing aids or cochlear implants.

No	Children’s use of hearing aids or cochlear implants	
	Hearing aid	Cochlear implant
C1		X **(Bilateral)**
C2	X **(Bilateral)**	
C3	X **(Bilateral)**	
C4	X **(Bilateral)**	
C5		X **(Bilateral)**
C6		X **(Bilateral)**
C7	X **(Bilateral)**	
C8		X **(Bilateral)**
C9		X **(Bilateral)**
C10		X **(Bilateral)**
C11		X **(Bilateral)**
C12	X **(Bilateral)**	
C13		X **(Bilateral)**
C14		X **(Bilateral)**
C15	X **(Bilateral)**	
C16	X **(Bilateral)**	
C17		X **(Bilateral)**
C18		X **(Bilateral)**
C19		X **(Bilateral)**
C20	X **(Bilateral)**	

Information regarding hearing aid use in children is as follows: Children with the codes C2-C3-C4-C7-C12-C15-C16-C20 use hearing aids, while the other children (C1-C5-C6-C8-C9-C10-C11-C13-C14-C17-C18-C19) use cochlear implants. Mothers have indicated that their children have hearing aids or bilateral cochlear implants. Children using bilateral cochlear implants are C1-C5-C6-C8-C9-C10-C11-C13-C14-C17-C18-C19. Children with codes C2-C3-C4-C7-C12-C15-C16-C20 use hearing aids, while the other children (C1-C5-C6-C8-C9-C10-C11-C13-C14-C17-C18-C19) use cochlear implants. It is understood that children generally start using hearing aids at the age of 1 after early diagnosis. When mothers were asked whether they used unilateral or bilateral hearing aids, this situation was determined.

As a result of the data analysis, the findings consisted of four main themes and 16 sub-themes. In the following section, first, demographic information, and then the main and sub-themes, were presented based on the answers to open-ended questions. This study was completed with the principle of volunteer participation in the phenomenological design in the qualitative research method. The researchers provided some explanations to the mothers before the interviews. They committed to not sharing data regarding children and families with any person or institution/organization, in accordance with confidentiality and ethical rules. Participants were informed that they had the right to withdraw from the interview at any time, including during the interview. The interview form was prepared by consulting one (1) expert in the field of hearing speech disorders and one (1) expert in the field of early childhood special education. The necessary ethical permissions for the research were obtained from the Hasan Kalyoncu University Scientific Research and Publication University Ethics Committee. The interviews took approximately 10–15 min with each mother, and the interview data were recorded in writing by the third researcher. According to the demographic information on families shown in Table 1, both mothers’ and fathers’ educational levels were similar at the primary-secondary level. None of the mothers had an active professional life, and half of them reported perceiving their socioeconomic status as “average.” Only M10, M12, M13, and M16 reported a good socioeconomic level. They stated that the family’s support came from their husbands and other family members (M1-M5-M9-M10). M2-M3 stated that they did not have support in their families. Mothers reported using sources recommended by teachers, such as storybooks, cartoons, and toys, to support their deaf children’s education. Special Education Institution: an education institution responsible for special education support services under the name of the Special Education and Rehabilitation Center, Language, Speech, and Communication Skill: LSC.

As shown in Table 2, 6 of the 20 children aged 4–5 years are female, and the others are male. To the question “Does your child have any other disabilities besides being deaf?” all mothers responded “No.” The communication model preferred in the family in daily life is sign language + verbal communication. M12-M13-M14-M15-M16 answered that their deaf children were on regular medication. It is noted that M10 has only one child, whereas M13 has seven. According to the mothers, the fields that should be supported most intensely are language, speech, and communication skills. There is deafness in the cousins of children of M5 and M18, in the siblings of children of M7, M8, M17, M19, and M20, and in the siblings of the husband (two uncles) of M10. Mothers have indicated that their children have hearing aids or bilateral cochlear implants. Children using bilateral cochlear implants are C1-C5-C6-C8-C9-C10-C11-C13-C14-C17-C18-C19. Children with codes C2-C3-C4-C7-C12-C15-C16-C20 use hearing aids, while the other children (C1-C5-C6-C8-C9-C10-C11-C13-C14-C17-C18-C19) use cochlear implants. It is understood that children generally start using hearing aids at the age of 1 after early diagnosis. When mothers were asked whether they used unilateral or bilateral hearing aids, this situation was determined. Information collected from mothers about their children’s hearing aid use is shown in Table 3. Regarding children’s use of hearing aids and cochlear implants, mothers reported that children mostly used them at 1.5–2 years of age.

## Results

Based on 20 mothers’ opinions, the findings of this study consisted of four main themes: *realization and guidance, family life changing with diagnosis and search for empowerment, holistic support for deaf children, sustainable and accessible support mechanisms,* and sub-themes related to these themes (Table 4: Mothers who expressed their opinions on the main and sub-themes). The participant mothers who stated opinions were assigned codes and numbers (e.g., M2 and M5). In the *Realization and guidance* main theme, for example, 12 participant mothers (M1-M2-M3-M4-M5-M8-M11-M12-M14-M18-M19-M20) stated opinions on the first theme, the need for information. Within the same main theme, mothers expressed opinions across multiple sub-themes. The main themes and sub-themes related to these themes are shown in Table 5.

**Table 4:** Mothers who expressed their opinions on the main and sub-themes.

Main themes	Mothers who expressed their opinions on the main and sub-themes
Realization and guidance	Need for information (M1-M2-M3-M4-M5-M8-M11-M12-M14-M18-M19-M20)
	Newborn Hearing Screening Test (M1-M5-M7-M8-M10-M11-M13-M15-M16-M17-M18-M19-M20)
	Mother-baby interaction (M3-M4-M6-M7-M8-M9-M12-M14)
	Guidance (M1-M5-M9-M12-M14-M17-M18-M19-M20)
	Late diagnosis (M2-M4-M8-M11-M14-M15)
Family life is changing with a diagnosis and a search for empowerment	Monitoring and supporting the development(M1-M2-M3-M4-M5-M6-M7-M8-M9-M10-M11-M12-M13-M14-M15-M16-M17-M18-M19-M20)
	Children’s and families’ needs shaped by the diagnosis ((M1-M2-M3-M4-M5-M6-M7-M8-M9-M10-M11-M12-M13-M14-M15-M16-M17-M18-M19-M20)
	Sources of support (M1-M4-M5-M7-M8-M9-M11-M12-M13-M14-M15-M16-M17-M18-M19-M20)
	Access to support and barriers to access(M1-M2-M3-M4-M5-M6-M7-M8-M9-M10-M11-M12-M13-M14-M15-M16-M17-M18-M19-M20)
Holistic support for deaf children	Early Childhood (M1-M2-M3-M8-M9-M11-M12-M13-M14-M15-M16-M17-M18-M19-M20)
	Preschool and school period (M1-M2-M3-M8-M9-M12-M13-M14-M15-M16-M17-M18-M19-M20)
	Social skills and participation in life (M1-M2-M3-M4-M5-M6-M7-M8-M9-M10-M11-M12-M13-M14-M15-M16-M17-M18-M19-M20)
	Opportunities (M1-M2-M3-M4-M5-M6-M7-M8-M9-M10-M11-M12-M13-M14-M15-M16-M17-M18-M19-M20)
Sustainable and accessible support mechanisms	Medical support, early diagnosis, and hearing aid implementation (M1-M2-M3-M4-M5-M6-M7-M8-M9-M10-M11-M12-M13-M14-M15-M16-M17-M18-M19-M20)
	Access to education and staff (M1-M2-M3-M4-M5-M6-M7-M8-M9-M10-M11-M12-M13-M14-M15-M16-M17-M18-M19-M20)
	Family’s place in sustainable policies for deaf children (M1-M2-M3-M4-M5-M6-M7-M8-M9-M10-M11-M12-M13-M14-M15-M16-M17-M18-M19-M20).

**Table 5:** Main themes and sub-themes created according to mothers’ opinions.

Main themes	Sub-themes
Realization and guidance	Need for information Newborn hearing screening test Mother-baby interaction Guidance Late diagnosis
Family life changing with the diagnosis and the search for empowerment	Monitoring and supporting the development Children’s and families’ needs shaped by the diagnosis Sources of support Access to support and barriers to access
Holistic support for deaf children	Early childhood Preschool and school period Social skills and participation in life Opportunities
Sustainable and accessible support mechanisms	Medical support, early diagnosis, and hearing aid implementation Access to education and staff Family’s place in sustainable policies for deaf children

### Main theme: realization and guidance

This main theme consists of five sub-themes.

#### Need for information

The mothers answered the questions, “What is deaf? How should deaf children be supported, what is expected for the family and child before and after the diagnosis?” Accordingly, the need for families to have information becomes prominent. Some examples of mothers’ opinions are as follows: *M3: As a mother, I knew there was something, but I was not an ENT doctor. M5: I realized that when my child could not pass the test, when he was born, and I needed direct information. I was informed, but I also consulted an acquaintance. M8: We already went to the doctor several times when the child did not talk. We did not receive any support from anyone. We had a test done, but we did not receive a clear result.*

#### Newborn hearing screening test

The majority of mothers emphasized the importance of the newborn hearing screening test and its impact on early diagnosis. For example, *M7* said *he failed the first test, as his elder brother had also failed it. He had a hearing aid implementation at the age of 1.5–2. M11* said *he passed the infancy period test, but we realized later. M16* said *it was congenital, he failed the test, and the process started immediately. M19* said *He failed the newborn screening, they guided us, and he was diagnosed thanks to other tests* and drew attention to the systematic implementation of newborn hearing tests in early diagnosis and intervention for deaf children.

#### Mother-baby interaction

Mothers reported that interaction patterns with their children vary at home, and they are concerned when their children are unresponsive to sounds and their own names. For example, *M3* said, *“We were calling the child’s name, but he was not looking at us.” He was not hearing our voices. He was constantly looking for someone with his eyes. M8, I realized it when she was 7 months old. She was not responding to sounds. She failed the test, and then they issued a diagnosis. M9 She was unresponsive to the sounds and staring blankly when she was 3.5 months old, I realized.* They stated that mother-baby interaction is frequently on families’ agendas regarding whether there is deafness in the early period. They stated that it is effective in receiving early diagnosis.

#### Guidance

Another sub-theme is how mothers suspect hearing or their processes for seeking a specialist doctor following a dialogue with someone in their immediate environment. Accordingly, mothers stated opinions as follows: *M12 We realized that he did not hear at home, and we took him to a doctor. We received the most support from doctors and our family. M14 when his speech decreased at home, we thought he was acting up. We took him to a psychiatrist, and he directed us to the department of ear, nose, and throat. Thus, we realized that he had a hearing problem. M19 The health workers in the hospital where I gave birth guided us.*

#### Late diagnosis

The mothers reported difficulties with guidance regarding late diagnosis in this sub-theme. They stated that their babies did not need an evaluation after the newborn screening test. Although this approach is effective for determining the severity of congenital deafness, it negatively affects follow-up to identify potential causes of later-onset deafness. The mothers stated that there could be delays in routine control applications. They stated that this process results in wasted time and delays early diagnosis. For example, *M8* said *she passed the newborn test at first. Thus, we clarified it after detailed tests, but we were a little late. M11* said *he passed the infancy period tests. We realized it later* and stated their opinions about the services they received in the field of health.

#### Main theme: family life changing with diagnosis and the search for empowerment

In this main theme, mothers emphasized the needs in their own and their children’s lives following the diagnosis. They mentioned support mechanisms and cooperation regarding the child’s and family’s needs, shaped by the diagnosis.

#### Monitoring and supporting the development

*M15* said. *We went to a university hospital with the guidance of a doctor at a private hospital that we went to; thus, the process was accelerated. We received a lot of support from the doctors. M7* said *he failed the first test, too, as did his elder brother. He had a hearing aid implementation at the age of 1.5–2. So we suspected. He was responsive only to loud sounds. We willingly moved forward, M9* said. *In the development of my daughter, the guidance of ENT doctors and the Rehabilitation Center contributed significantly* and highlighted the importance of monitoring development with the diagnosis and the necessity of supporting game skills.

#### Children’s and families’ needs shaped by the diagnosis

Mothers reported that they mostly need information, psychological and financial support, toys, materials, and educational resources for themselves and their children. For example, *M2, the mother, needs a lot of patience. It is such a difficult process. They need a lot of attention and compassion. In the end, we need patience and guidance on how to help our children talk better. M4, I did not know how to support him in understanding instructions and speech or in responding to sounds. I had financial problems. I needed financial support.*

#### Sources of support

The mothers who participated in the study stated that they most frequently received support from the health institution, authorities at the special education institution, educators, and their own family members (M2, M3, and M6 participating mothers did not support this view). For example, *M1* said *I always consulted about how to communicate for her to be able to say her needs, such as playing games, water or food, financial needs may occur, to be able to repair the hearing aid when it breaks down, or buy a new one when it is lost. M12* said *I receive support from my family and my staff at the educational institution.*

#### Access to support and barriers to access

Mothers drew attention to barriers to accessing supports and to their children’s ability to benefit from education and health services on equal terms with their peers. They mentioned the family’s financial status, difficulties accessing experts for early diagnosis, delays in diagnosis, late implementation of hearing aids, delays in the treatment process, and the use of assistive technology. For example, *M7* said *preschool is a period that requires more attention; it is the stage of exploring the environment. The early period is important for children to receive quality education in special education since it is necessary to meet their needs before school. ... M9* said, *We and our children want to be understood in society; financial freedom is also important, and family support and social support should be provided for children between the ages of 0 and 6 (the age group of our children).*

### Main theme: holistic support for deaf children

In this theme, mothers expressed a strong need for early medical diagnosis and early intervention services. They emphasized the importance of proper rehabilitation services in the preschool period. They also emphasized the importance of community support for daily living and social skill development.

#### Early childhood

The majority of mothers emphasized that the early childhood period is critical and that children must benefit from early education, with early diagnosis and intervention. For example, *M14* said *education support and attention are needed. Families should answer children’s questions adequately for their benefit.*

#### Preschool and school period

The mothers drew attention to the preparation of preschool skills and stated that they think monitoring and support should be tailored to the needs during the school period. For example, *M9* said *that preschool education should definitely be provided for at least 2 years. The children must progress in expressing themselves and communicating in the family until the preschool period. M16* said *the child must have the habit of constantly using the hearing aid. They should attend rehabilitation institutions regularly.*

#### Social skills and participation in life

Among mothers, while M10-M17-M18-M19 emphasized the importance of special education services and inter-family support in the child’s independent life skills from an early age, other mothers emphasized opportunities that strengthen social adaptation and peer interaction. In this regard, they reported that the participation of deaf children with their typically hearing peers should be given importance.

#### Opportunities

Mothers reported that they consider receiving early diagnosis and accessing early childhood special education services as opportunities. They drew attention to the need for educational institutions to provide quality education and expressed opinions on the effects of family education programs. For example, while M3-M11-M15-M16 suggested a program based on special education and individual support, M4-M5-M6-M7 stated that personnel in the institutions and learning opportunities are insufficient.

#### Main theme: sustainable and accessible support mechanisms

In the last main theme, mothers emphasized medical support, access to education, and cooperation with personnel. They stated that cooperation should be sustainable and suggested that family participation should be an important component of legal policies.

#### Medical support, early diagnosis, and hearing aid implementation

For example, in the first theme, mothers stated that medical support should continue with family participation after the newborn screening. They emphasized the importance of hearing screening during childhood. *M3* said *it is necessary not to disrupt family education and rehabilitation education, and to revise at home.*

#### Access to education and staff

In this sub-theme, which focuses on the right to education and access, mothers emphasized inclusive education, called for peers to be informed, and highlighted the importance of family participation. *M4* said *it should be suggested that IEP plans be implemented effectively. Support should be provided to address all developmental needs after the diagnosis; cooperation with teachers should be encouraged; teachers should not change frequently; peer bullying should be prevented in schools;* and attention should be paid to the need for inclusive educational environments. *M9* stated her opinion by saying, “*It is important for families to participate in education to provide an environment where the children can integrate with their classmates by realizing that deaf children do not have a mental deficiency; on the contrary, their potential should be realized.”*

#### Family’s place in sustainable policies for deaf children

Mothers, expressing their opinions on education and health policies, emphasized that negative comments should not be made about the effort families make for their children at school and that children should typically be informed to foster an inclusive education environment. For example, *M15* said *she wants to provide security for her children while they are at school. Besides, if the classroom teacher seats her in the front row, she will not have difficulty hearing, and if the teacher informs her classmates about her situation, that would be good.*

In conclusion, mothers drew attention to both medical and educational evaluations; they also emphasized the importance of early diagnosis and education in health, education, and social rights across the main themes and sub-themes. Regarding the realization of the family and access to support, they stated that the need for information should be met. They shared that families need financial and social support and emphasized the importance of expanding the scope of newborn screening and monitoring studies.

There are several important points to consider when discussing the results of this study. For example, whether children have hearing aids or cochlear implants, the family’s needs and dynamics, such as socioeconomic status, whether the child received an early diagnosis, and access to and participation in support services are all important factors. Findings related to both children with hearing loss and their mothers were discussed in this context.

## Discussion

In our study, we aimed to reveal the processes and difficulties experienced by deaf children from birth through early childhood services, as perceived by their mothers. The findings from the interviews we conducted with mothers are discussed in the literature below. The first theme is *“Realization and guidance.”* Within the scope of this theme, mothers stated that implementing NNHSP is important for early diagnosis and increased awareness among children. The literature states that the benefits of implementing NNHSP are clearly appreciable and that it reduces the age of deafness detection in the early stages. Thus, it is stated that there are developments in early intervention (Wroblewska-Seniuk et al., 2017). In our study, mothers who stated they needed information after early diagnosis primarily expressed a need for information on interaction and communication with their children. They expressed a desire to receive applied information on how to communicate with their children. In our study, we found that mothers did not practice newborn hearing screening when they did not give birth in a hospital environment, or there were delays in intervention, because they did not know how to proceed after these tests. Although the practice of NNHSP is widely used throughout the country (Kemaloğlu, 2015), the study’s results may be explained by the fact that the region where the study was conducted was a rural area with low socioeconomic status and educational levels. Olusanya et al. (2004) stated that it is difficult to identify newborn babies with mild deafness and deaf children with delayed onset. Moreover, while deafness can be detected through hearing screening, infants born at home rather than in a hospital can be identified later (Yoshinaga-Itano, 2003). Thus, in our study, we believe that difficulties in identifying deafness stem from factors such as families’ inability to access health services in rural areas and the late onset of the condition.

Within the scope of the study, it draws attention to the fact that the majority of mothers need information about the appropriate hearing aid, health controls, and deafness from the diagnosis process of their children. Moreover, they underlined their need for guidance for monitoring their children’s development after birth and diagnosis. They expressed the importance of guidance and receiving information. In addition to all of these, they stated that they need support in meeting their basic needs, including psychological, financial, educational, and health needs. As stated in the literature, families experience highly overwhelming and difficult processes regarding the evaluation and diagnosis of deafness and the implementation of hearing aids during infancy (Rice and Lenihan, 2005). Families typically develop emotions such as shock, denial, and acceptance in these processes. During critical periods, they are expected to make important decisions about interventions for their children as early as possible (Matthijs et al., 2012). The important decisions made during the implementation of hearing aids or cochlear implants for deaf children affect their development. Moreover, families need to decide on communication methods with their children in the early period and plan accordingly (Decker et al., 2012). This situation is supported by Matthijs et al. (2012). When considering the family structures, number of children, socioeconomic status, educational levels, and active participation in professional life, the mothers we interviewed in this study need a high level of financial, psychological, and educational and health support. The mothers stated that they need monitoring, guidance, and support for how to proceed with their children after the diagnosis and from whom to receive support for these steps. In this regard, Gilliver et al. (2013) reported that families’ emotions were mixed after the diagnosis of deafness, and they received support from experts, the internet, and their friends during this period. In addition, many families stated they were unable to receive sufficient support from the experts. Similarly, families in our study mentioned that early diagnosis is important for early intervention. Although these practices are developing in Türkiye, it is thought that these services should become more widespread, based on factors such as the family’s situation, the environment in which they live, and the characteristics of their family structure. The study’s findings show that mothers stated that support services should begin in early childhood and continue through preschool and school. They emphasized that these regulations will strengthen deaf children’s social, language, and communication skills and enable them to participate in daily life. This finding was similarly supported in the literature. For example, it is stated that families do not have information about the communication method they will choose for the education services to be provided after early diagnosis and the implementation of appropriate hearing aids, and they experience difficulties (Eleweke and Rodda, 2000; Roberts et al., 2015). Decker et al. (2012) stated in their study with families of deaf children that their communication preferences, whether verbal or sign language, are influenced by the education they received from experts and others in their environment. While families who receive support from language and speech therapists or audiologists tend to use verbal language, they stated that they prefer sign language because of their friends, environment, and relatives. In our study, similarly, mothers seek support from experts to develop their children’s language, communication, and social skills. In the study’s findings, mothers lastly noted that support services should be sustainable and that cooperation with experts is needed from the children’s diagnosis stage onward. They stated that, thus, they would not walk alone, and access to education and health services would be easier. Regarding family and expert cooperation, Bakker and Denessen (2007) noted that teachers and experts should possess attitudes, knowledge, and experience that foster cooperation with families in successful educational services. They emphasized that cooperation between home and school is highly difficult but extremely important. Turnbull et al. (2015) stated that cooperation with families must be addressed holistically; support should be provided not only at the school level but also at all stages of development, from the children’s diagnosis of deafness. The literature on this subject highlights the need for families and experts to holistically collaborate at all stages of deaf children’s development, from diagnosis. However, in cooperation, experts and educators should be well-rounded in their attitudes toward families, as well as in their approaches, knowledge, and skills. Thus, families can more easily cope with the difficulties resulting from their children’s deafness from an early age and support them in a positive environment (Mapp et al., 2014; Todd et al., 2014). In our study, similar findings emerged: families do not holistically collaborate with experts and lack knowledge and experience in caring for their children.

### Limitations

The limitations of this study are as follows. The study was conducted with 20 parents using a qualitative research method. All participants were mothers, and all children were deaf and diagnosed in early childhood. The study focused on the preschool processes of deaf children and is therefore limited to the preparation phase from home to school, as well as parents’ opinions and experiences. A semi-structured interview form was utilized during the data collection process. Additionally, no observations of the school environment and/or the home/institution were conducted.

Suggestions regarding the field;

- Early childhood family training can be provided to support deaf children’s school-readiness skills.
- Training manuals can be prepared for the child’s parents on the school readiness process, including laws, school rules, and the parental role in academic and social participation.
- It may be recommended to raise awareness and provide sign language training to parents.

Regarding further research;

- A study can be planned with fathers, siblings, and/or experts who provide educational, training, and health services to deaf children.
- A longitudinal study can be conducted to collect data on deaf children’s access to preschool education and support for their academic and social skills, communication, language, and speech during the school period.
- Studies can be planned based on the early childhood experiences of deaf adults or individuals in secondary and higher education.
- Studies can be designed using quantitative, qualitative, or mixed methods. Studies can be designed based on families’ opinions on health and education regulations.
- Studies can be planned based on families’ experiences with their children’s literacy, behavior, language, and communication skills.

## Conclusion

In conclusion, our study highlighted the importance of early diagnosis for the early implementation of hearing aids and education. It revealed that families who realize deafness late or do not know how to proceed after diagnosis cannot benefit from these interventions. Another important result of our study is the difficulty in providing families with support in implementing hearing aids and communication methods for children diagnosed early. The families drew attention to the fact that the most accurate information and guidance about their children should be provided in a developmental process. Moreover, they mentioned that the support provided by the experts should include early education services, and they emphasized the importance of cooperation for these supports. In line with these results, the development of deaf children should be followed not only during the diagnostic stage but also holistically by the official institutions responsible for health, education, and social services. Thus, it is thought that children’s participation in daily life and the development of their language and communication skills will be achieved more effectively by fostering cooperation between families and experts and by providing families with support in the areas they need.

## Data Availability

The original contributions presented in the study are included in the article/Supplementary material, further inquiries can be directed to the corresponding author.
