# Supplementary material for: Mothers’ views on the school-starting process for deaf children
Source: Front Psychol. 2026 Jan 8;16:1694936. doi: 10.3389/fpsyg.2025.1694936 (PMC12823789; doi:10.3389/fpsyg.2025.1694936)
Supplement: Supplementary file 1 [file Supplementary_file_1.docx]

**Appendix 1**

**Demographıc Informatıon Form for Deaf Chıldren**

Interview Date:

1. Date of Birth of your child: Gender: Girl ( ) Boy ( )
2. Age-month?
3. Does he/she have any other disabilities other than hearing loss? If yes, please write briefly
4. Are there any other individuals with hearing loss in your family? If yes, who/who are they?
5. Who/whom did you receive support from in your child's early childhood. Could you briefly explain these supports?
6. What is the communication model used in the family or preferred in daily communication?

Sign language ( ) Verbal Communication ( ) Sign language + Verbal Communication ( )

1. How many children do you have other than your child with hearing loss?

1 ( ) 2 ( ) 3 ( ) 4 ( ) Other................

1. Which of the following does your child use?

Hearing aid ( ) Cochlear implant ( ) Hearing aid+Cochlear implant ( ) Not using ( )

1. Does your child use the device regularly? Yes ( ) No ( )
2. Hearing Aid or Cochlear Implant Use Status: Bilateral ( ) Unilateral ( )
3. Does your child receive special education support?
4. What are the areas where you think your child needs the most intensive support? Please write... (For example academic skills, behavioral support, language, speech, social skills, communication skills, etc.....)
5. Are there any medicines that you take continuously? Yes ( ) No ( ) If yes, please write their names?

**Appendix 2- Informatıon Form for Parents of Deaf Chıldren**

**Dear parent, you can answer the questions below as a participating parent. Thank you very much for your support**

1. Age of mother Education Level: Are you actively working in a job?
2. Age of father Education Level: Are you actively working in a job?
3. Who/whom did you receive the most intensive support from when you needed support for your child?
4. What are the sources you most frequently consult for information material support on hearing loss?
5. What (in your
6. opinion) is the perceived socio-economic status of your family

**Appendix. 3 Semı-Structured Intervıew Form**

1. Can you tell us about the assessment and diagnostic history of your child's deafness?

a) How was it recognized? Who/whom did you receive the most intense support during the referral process to the relevant health institution?

2. What kind of needs do you think deaf children and their families have?

3. Who/what are the people/resources you consulted about your child in early childhood? What kind of support do you receive from these sources?

4. What do you think deaf children’s school readiness skills are? How should children be supported in school readiness processes?

5. What kind of planning did you do before starting pre-school education? What were the subjects you wanted to be informed about most frequently in this process? Why?

6. What are your suggestions for supporting deaf children's school readiness skills?

a) To families of deaf children b) School administrators c) Teachers d) Peers e) Families of hearing children

| **No** | **Mother's Education** | **Father's Education** | **Mother's Age** | **Father's Age** | **Father's Profession** | **Family's Source of Support** | **Source Regarding D** | **Perceived SEL** |
| --- | --- | --- | --- | --- | --- | --- | --- | --- |
| M1 | Secondary School | High School | 26 | 30 | Employee | Husband and family members | Toy | Average |
| M2 | High School | High School | 31 | 35 | Employee | No support | Education institution | Average |
| M3 | Secondary School | Secondary School | 26 | 32 | Employee | No support | Audiologist, hearing aidcompany | Average |
| M4 | Illiterate | Primary School | 35 | 39 | Employee | Education institution | Education institution | Low |
| M5 | Primary School | Primary School | 26 | 35 | Technician | Family, education institution | Education institution, toy | Low |
| M6 | Illiterate | Primary School | 35 | 42 | Employee | Family | Internet, hospital | Low |
| M7 | Primary School | Secondary School | 29 | 39 | Employee | Husband | Hospitals, education institutions | Low |
| M8 | High School | High School | 43 | 58 | Retired | Special education teacher | Education institution | Low |
| M9 | University | University | 35 | 38 | Doctor | Grandmother | Toy, book | Average |
| M10 | Secondary School | Secondary School | 22 | 27 | Employee | Family, hospital staff | Education institution, toy | Good |
| M11 | Primary School | Secondary School | 48 | 51 | Employee | No | Teacher, book | Low |
| M12 | Primary School | Secondary School | 30 | 32 | Farming | Family, education institution | Teacher, expert | Good |
| M13 | Primary School | Secondary School | 40 | 45 | Driver | No support | Internet, books | Good |
| M14 | High School | High School | 38 | 38 | Employee | Education institution | Toy, books | Average |
| M15 | Primary School | High School | 32 | 35 | Employee | Education institution | Educational material | Average |
| M16 | High School | High School | 27 | 34 | Employee | Husband, mother | Teacher, expert | Good |
| M17 | Secondary School | Secondary School | 28 | 32 | Employee | Education institution | Story, cartoon | Average |
| M18 | Secondary School | Secondary School | 24 | 32 | Furnisher | Grandmother, grandfather | Internet, experts | Average |
| M19 | Secondary School | Secondary School | 28 | 32 | Employee | Husband, education institution | Storybooks, cartoon | Average |
| M20 | Primary School | High School | 43 | 40 | Driver | Education institution | Stories, toys | Average |

**Table-1. Demographic Information Regarding the Families**

**Education Institution:** Special Education and Rehabilitation Center **M:** Mother **SEL:** Socioeconomic Level

| **Table-2 Demographic Information Regarding Deaf Children (Information was obtained through interviews with mothers)** | | | | | | |
| --- | --- | --- | --- | --- | --- | --- |
| **Code** | **Child Gender/Age** | | **Mother's Source of Support** | **Family's Number of Children**  **(except D)** | **Support Needs of Deaf Child** | |
| M1 | Female | 4-year-old | Husband | 2 | LSC |  |
| M2 | Male | 4-year-old | No Source of Support | 1 | LSC |  |
| M3 | Male | 4-year-old | No Source of Support | 2 | LSC, preparation to school |  |
| M4 | Male | 4 years 6 months | SEI | 4 | LSC |  |
| M5 | Male | 5-year-old | SEI | 2 | LSC, peer interaction |  |
| M6 | Male | 4 years 4 months | No Source of Support | 1 | LSC, game skills |  |
| M7 | Male | 4 years 11 months | Hospital, Special Edu. Tchr. | 1 | LSC |  |
| M8 | Female | 5-year-old | Special Edu. Tchr. | 1 | LSC |  |
| M9 | Female | 5-year-old | SEI | 1 | LSC |  |
| M10 | Male | 4 years 11 months | Own family members | 0 | LSC |  |
| M11 | Male | 5-year-old | No | 4 | LSC |  |
| M12 | Male | 4 years 11 months | SEI | 2 | LSC |  |
| M13 | Female | 4 years 2 months | SEI | 6 | LSC |  |
| M14 | Male | 5-year-old | SEI | 2 | LSC |  |
| M15 | Female | 5 years 4 months | SEI | 1 | LSC |  |
| M16 | Male | 5 years 3 months | SEI | 1 | LSC |  |
| M17 | Male | 5 years 1 month | SEI | 1 | LSC |  |
| M18 | Male | 5 years 6 months | Husband, family members | 1 | LSC |  |
| M19 | Male | 5-year-old | Teacher | 1 | LSC |  |
| M20 | Female | 5 years 5 months | Doctor, SEI | 3 | Academic skills, LSC |  |

**Special Education Institution:** education institution responsible for special education support services under the name of special education and rehabilitation center, **Language, Speech, and Communication Skill:** LSC

When examining the support resources of mothers whose children are mostly between the ages of 4 and 5, it is noteworthy that they receive support from special education and rehabilitation support centers. The support needed is focused on their children's language skills.

**Table-3 Children's use of hearing aids or cochlear implants**

|  | **Children's use of hearing aids or cochlear implants** | |
| --- | --- | --- |
| **No** | **Hearing Aid** | **Cochlear Implant** |
| C1 |  | X **(Bilateral)** |
| C2 | X **(Bilateral)** |  |
| C3 | X **(Bilateral)** |  |
| C4 | X **(Bilateral)** |  |
| C5 |  | X **(Bilateral)** |
| C6 |  | X **(Bilateral)** |
| C7 | X **(Bilateral)** |  |
| C8 |  | X **(Bilateral)** |
| C9 |  | X **(Bilateral)** |
| C10 |  | X **(Bilateral)** |
| C11 |  | X **(Bilateral)** |
| C12 | X  **(Bilateral)** |  |
| C13 |  | X **(Bilateral)** |
| C14 |  | X **(Bilateral)** |
| C15 | X **(Bilateral)** |  |
| C16 | X **(Bilateral)** |  |
| C17 |  | X **(Bilateral)** |
| C18 |  | X **(Bilateral)** |
| C19 |  | X **(Bilateral)** |
| C20 | X **(Bilateral)** |  |

Information regarding hearing aid use in children is as follows:

Children with the codes C2-C3-C4-C7-C12-C15-C16-C20 use hearing aids, while the other children(C1-C5-C6-C8-C9-C10-C11-C13-C14-C17-C18-C19) use cochlear implants. Mothers have indicated that their children have hearing aids or bilateral cochlear implants. Children using bilateral cochlear implants are C1-C5-C6-C8-C9-C10-C11-C13-C14-C17-C18-C19. Children with codes C2-C3-C4-C7-C12-C15-C16-C20 use hearing aids, while the other children (C1-C5-C6-C8-C9-C10-C11-C13-C14-C17-C18-C19) use cochlear implants. It is understood that children generally start using hearing aids at the age of 1 after early diagnosis. When mothers were asked whether they used unilateral or bilateral hearing aids, this situation was determined.

**Table 4-** **Mothers who expressed their opinions on the main and sub-themes**

| **Main Themes** | **Mothers who expressed their opinions on the main and sub-themes** |
| --- | --- |
| Realization and guidance | Need for information (M1-M2-M3-M4-M5-M8-M11-M12-M14-M18-M19-M20)  Newborn Hearing Screening Test (M1-M5-M7-M8-M10-M11-M13-M15-M16-M17-M18-M19-M20)  Mother-baby interaction (M3-M4-M6-M7-M8-M9-M12-M14)  Guidance (M1-M5-M9-M12-M14-M17-M18-M19-M20)  Late diagnosis (M2-M4-M8-M11-M14-M15) |
| Family life changing with diagnosis and search for empowerment | Monitoring and supporting the development(M1-M2-M3-M4-M5-M6-M7-M8-M9-M10-M11-M12-M13-M14-M15-M16-M17-M18-M19-M20)  Children’s and families’ needs shaped by the diagnosis (M1-M2-M3-M4-M5-M6-M7-M8-M9-M10-M11-M12-M13-M14-M15-M16-M17-M18-M19-M20)  Sources of support (M1-M4-M5-M7-M8-M9-M11-M12-M13-M14-M15-M16-M17-M18-M19-M20)  Access to support and barriers to access (M1-M2-M3-M4-M5-M6-M7-M8-M9-M10-M11-M12-M13-M14-M15-M16-M17-M18-M19-M20) |
| Holistic support for deaf children | Early Childhood (M1-M2-M3-M8-M9-M11-M12-M13-M14-M15-M16-M17-M18-M19-M20)  Preschool and school period (M1-M2-M3-M8-M9-M12-M13-M14-M15-M16-M17-M18-M19-M20)  Social skills and participation in life (M1-M2-M3-M4-M5-M6-M7-M8-M9-M10-M11-M12-M13-M14-M15-M16-M17-M18-M19-M20)  Opportunities (M1-M2-M3-M4-M5-M6-M7-M8-M9-M10-M11-M12-M13-M14-M15-M16-M17-M18-M19-M20) |
| Sustainable and accessible support mechanisms | Medical support, early diagnosis, and hearing aid implementation (M1-M2-M3-M4-M5-M6-M7-M8-M9-M10-M11-M12-M13-M14-M15-M16-M17-M18-M19-M20)  Access to education and staff (M1-M2-M3-M4-M5-M6-M7-M8-M9-M10-M11-M12-M13-M14-M15-M16-M17-M18-M19-M20)  Family's place in sustainable policies for deaf children (M1-M2-M3-M4-M5-M6-M7-M8-M9-M10-M11-M12-M13-M14-M15-M16-M17-M18-M19-M20). |

**TABLE 5 Main themes and sub-themes created according to mothers’ opinions.**

| **Main Themes** | **Sub-themes** |
| --- | --- |
| Realization and guidance | - Need for Information - Newborn Hearing Screening Test - Mother-baby interaction - Guidance - Late diagnosis |
| Family life changing with diagnosis and search for empowerment | - Monitoring and supporting the development - Child's and family's needs shaped with the diagnosis - Sources of support - Access to support and barriers to access |
| Holistic support for deaf children | - Early childhood - Preschool and school period - Social skills and participation in life - Opportunities |
| Sustainable and accessible support mechanisms | - Medical support, early diagnosis, and hearing aid implementation - Access to education and staff - Family's place in sustainable policies for deaf children |
